# U. S. Naval Surgeons

**Published:** 1848-10

**Authors:** 


*U. S. Naval Surgeons.*—A Board of Naval Surgeons will assemble at Philadelphia, October 25th, for the examination of candidates for admission into the Navy as Assistant Surgeons. Persons who are twenty-one, and not over twenty-eight years old, desiring to appear before the Board, can receive permission by making application, accompanied by proper testimonials, to the Secretary of the Navy.—*Washington Union.*

